# Family-Partnered Delirium Care in the ICU: Feasible Today, Essential Tomorrow?

**DOI:** 10.1097/CCE.0000000000001318

**Published:** 2025-09-09

**Authors:** Vanessa Vater, Peter Nydahl, Pam Ramsay

**Affiliations:** 1 University Hospital of Frankfurt, Frankfurt, Germany.; 2 University Hospital of Schleswig-Holstein, Kiel, Germany.; 3 Institute of Nursing Science and Practice, Paracelsus Medical University, Salzburg, Austria.; 4 School of Health Sciences, University of Dundee, Scotland, United Kingdom.

**Keywords:** critical care communication, family engagement, family-centered care, humanizing critical care, visitation policies

Delirium in ICU patients remains a common and major clinical challenge and with a frequency of 19–82% represents an acute emergency (1, 2). It is associated with increased mortality and morbidity, worse functional and cognitive outcomes, and a higher rate of institutionalization after hospital discharge (2).

Patients experiencing delirium often suffer from fluctuating orientation, disturbances of consciousness, and impairments in memory and thinking. They may also experience hallucinations, delusional experiences, and nightmares. Nevertheless, they may remain aware of their surroundings and of the people around them, and they may be capable of perceiving the hospital environment and medical interventions (3).

In current best-practice approaches, management of delirium includes the use of validated tools for identification of the risk and incidence of delirium throughout the patient’s ICU stay. Underlying causes, such as infections, should be detected and treated, and treatment strategies should aim to minimize iatrogenic harm. Whenever possible, sedatives, opioids, and antipsychotics should be avoided. Nonpharmacological interventions include information and communication, mobilization, optimizing environmental factors, and integrating families (2, 4). Despite the publication of hundreds of studies in recent years on conceptual frameworks, models, and evidence-based strategies for the prevention and treatment of delirium—as well as the availability of validated assessment instruments—delirium management remains insufficient and as consequence, impacts therapy and patient outcomes.

The patient’s family is a valuable resource in the recognition of delirium, as family carers may be better able to recognize subtle changes in patient cognition and behavior (5). For many patients, the experience of delirium is associated with anxiety and shame (6). For family members, witnessing delirium is associated with feelings of anxiety, embarrassment, uncertainty, and shock (7). Therefore, caring for this patient population requires significant clinical knowledge, empathy, and compassion. Family involvement plays a crucial role, as a humanized and person-centered approach to delirium can enhance the ability of both family members and interdisciplinary teams to support patients through these challenges (8).

Unlike clinical staff, family members may be able to access patients emotionally and communicate with them more effectively. There are multiple approaches to integrating families into care delivery, including family visits, telephone or video calls, physical presence, and participation in nursing interventions and multidisciplinary ward rounds. Family involvement can function as an independent intervention or be integrated into care bundles such as the “ABCDEF bundle.”

The Society of Critical Care Medicine recommends family involvement to enhance the effectiveness of nonpharmacological such as reorientation, cognitive stimulation, and early mobilization for delirium prevention, even though the overall quality of evidence remains low (9). Several randomized controlled trials (RCTs) have demonstrated that family involvement can significantly reduce the prevalence of delirium in ICU patients (10). This represents an underutilized potential for expanding current delirium management strategies. In the midst of highly technical care and resource and educational constraints, a promising new approach is therefore emerging: the structured inclusion of family members in educational, detective, and preventive measures.

In this issue of *Critical Care Explorations*, the recently published Activating Family Caregivers in the Identification Prevention and Management of Delirium (ACTIVATE) study by Fiest et al (11) offers important new insights. This pilot RCT evaluates the feasibility and acceptability of a structured, family-partnered approach to delirium prevention, detection, and management in the ICU setting. The results of this pilot study shed new light on a long-standing problem. The multicenter ACTIVATE study by Fiest et al (11) enrolled 64 patient-family dyads at two Canadian ICUs. Family members were predominantly spouses or children. The intervention group received structured delirium education, a preventive intervention checklist, and the validated Sour Seven screening tool for detection (5). The control group received standard care and a general delirium information leaflet. Despite pandemic-related recruitment challenges, a 32% participation rate was achieved, and 75% of family members completed outcome measures for anxiety and depression. Notably, 42% of family members in the intervention group were able to correctly identify delirium using the Sour Seven.

The study emphasizes the potential of families to contribute meaningfully to patient care when they are informed, included, and valued as partners. ACTIVATE was conducted under the unique and restrictive conditions of the COVID-19 pandemic, a time during which family involvement was particularly limited. The fact that 64 dyads could still be enrolled highlights the outstanding engagement of the researchers and participating teams but shows also the urgent need for such involvement. The finding that delirium was rarely part of ward round communication underscores the persistent gap between knowledge and implementation and points to the potential of family members as communicative catalysts.

Most families want to be involved. While witnessing delirium is often distressing, families are too often relegated to passive roles. Such passivity can be constructively overcome. Family members can be integrated as a valuable resource in delirium detection, as they are often able to notice subtle changes in behavior and cognition more quickly than nursing staff. The Sour Seven has proven to be a practical tool in other studies (5, 12). The form and extent of family involvement may vary. For example, Johnson et al (13) demonstrated that audio recordings of family voices can provide orientation, offer comforting words, and reduce anxiety in patients with delirium. Rosa et al (14) showed that implementing flexible visiting hours in the ICU increased family presence, which, in turn, led to greater involvement in delirium prevention efforts. Wheeler et al (15) created both in-person and virtual options for families to learn about delirium and found that such education resulted in a measurable increase in delirium-related knowledge.

What distinguishes ACTIVATE from these earlier studies is the integration of multiple components: structured education, specific screening tools, delirium prevention checklists, and systematic evaluation within a RCT design. Earlier studies often focused on individual measures (e.g., visiting hours). ACTIVATE brings these elements together into a practical, methodologically robust concept, and for the first time provides evidence for the feasibility, acceptability, and potential efficacy of a multidimensional family engagement approach. Furthermore, the study addresses a frequently overlooked dimension: the impact of such interventions on families themselves. While many studies focus primarily on patient outcomes, ACTIVATE also incorporates the psychologic burden and lived experience of family members, of whom 12 reported clinically significant scores for anxiety or depression. This broader perspective aligns with current recommendations related to Post-Intensive Care Syndrome-Family and underscores the relevance of holistic models of care.

For clinical implementation, educational materials must be accessible, culturally sensitive, and seamlessly integrated into daily hospital routines. ICU staff should be trained not only in delivering such programs but also supported in partnering effectively with family members. In addition, institutional policies are needed that allow for flexible visitation without compromising clinical workflows or patient safety. ACTIVATE offers an adaptable framework for this purpose, but its sustainable implementation will require interdisciplinary collaboration. From a scientific perspective, larger multicenter RCTs are needed to assess the clinical effectiveness of family-partnered delirium programs on patient-centered outcomes such as delirium duration, ICU length of stay, or long-term cognitive function. Implementation research will be critical in identifying barriers, facilitators, and contextual factors that influence sustainable adoption. Future studies should also address economic considerations, staff impact, and the psychologic effects on both patients and families (**Fig. 1**).

In summary, the ACTIVATE study provides compelling evidence of feasibility, showing that family members can play a meaningful role in delirium care. ACTIVATE has opened a door toward a more human-centered and collaborative model of intensive care—one that is not only possible but essential.

**Figure 1.**
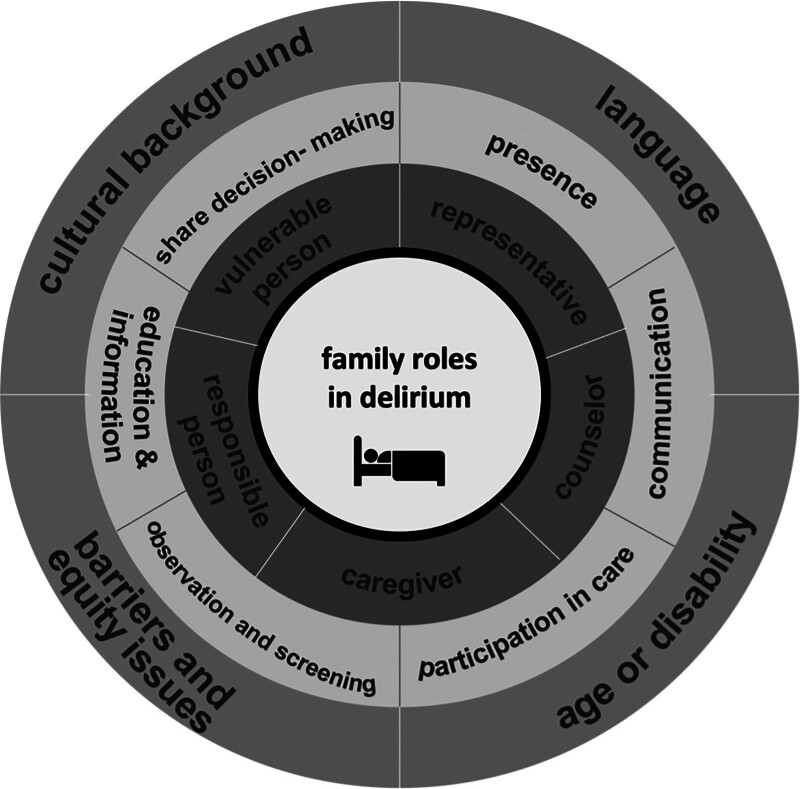
Family roles in delirium. From the inside out: roles of relatives, forms of involvement of relatives, barriers, and aspects of justice.

## References

[1] Hshieh TT Inouye SK Oh ES: Delirium in the elderly. Psychiatr Clin North Am 2018; 41:1–17 29412839 10.1016/j.psc.2017.10.001

[2] Wilson JE Mart MF Cunningham C: Delirium. Nat Rev Dis Primers 2020; 6:90 33184265 10.1038/s41572-020-00223-4 PMC9012267

[3] Li J Fan Y Luo R: Family involvement in preventing delirium in critically ill patients: A systematic review and meta-analysis. Int J Nurs Stud 2025; 161:104937 39486106 10.1016/j.ijnurstu.2024.104937

[4] Sosnowski K Lin F Chaboyer W: The effect of the ABCDE/ABCDEF bundle on delirium, functional outcomes, and quality of life in critically ill patients: A systematic review and meta-analysis. Int J Nurs Stud 2023; 138:104410 36577261 10.1016/j.ijnurstu.2022.104410

[5] Krewulak KD Sept BG Stelfox HT: Feasibility and acceptability of family administration of delirium detection tools in the intensive care unit: A patient-oriented pilot study. CMAJ Open 2019; 7:E294–E299 10.9778/cmajo.20180123 PMC6488481 31028053

[6] Boehm LM Jones AC Selim AA: Delirium-related distress in the ICU: A qualitative meta-synthesis of patient and family perspectives and experiences. Int J Nurs Stud 2021; 122:104030 34343884 10.1016/j.ijnurstu.2021.104030 PMC8440491

[7] Lange S Mȩdrzycka-Da Browska W Friganović A: Family experiences and attitudes toward care of ICU patients with delirium: A scoping review. Front Public Health 2022; 10:1060518 36505003 10.3389/fpubh.2022.1060518 PMC9727388

[8] Nydahl P Ely EW Heras-La Calle G: Humanizing delirium care. Intensive Care Med 2024; 50:469–471 38300266 10.1007/s00134-024-07329-3 PMC10954974

[9] Devlin JW Skrobik Y Gélinas C: Clinical practice guidelines for the prevention and management of pain, agitation/sedation, delirium, immobility, and sleep disruption in adult patients in the ICU. Crit Care Med 2018; 46:e825–e873 30113379 10.1097/CCM.0000000000003299

[10] Lv Y Li P Li R: The impact of patient- and family-centered care interventions on intensive care unit outcomes: A meta-analysis of randomized controlled trials. Braz J Anesthesiol 2024; 75:844577 39608600 10.1016/j.bjane.2024.844577 PMC11714723

[11] Fiest KM Krewulak KD Sept BG: A pilot randomized controlled trial assessing the feasibility and acceptability of family-partnered delirium prevention, detection, and management in critically ill adults: The Activating Family Caregivers in the Identification Prevention and Management of Delirium (ACTIVATE) study. Crit Care Explor 2025; 7:e1287 40844707 10.1097/CCE.0000000000001287 PMC12377300

[12] Krewulak KD Bull MJ Wesley Ely E: Effectiveness of an intensive care unit family education intervention on delirium knowledge: A pre-test post-test quasi-experimental study. Can J Anaesth 2020; 67:1761–1774 32959203 10.1007/s12630-020-01810-5 PMC7716844

[13] Johnson GU Towell-Barnard A McLean C: The development of a family-led novel intervention for delirium prevention and management in the adult intensive care unit: A co-design qualitative study. Aust Crit Care 2025; 38:101088 39129064 10.1016/j.aucc.2024.07.076

[14] Rosa RG Falavigna M da Silva DB; ICU Visits Study Group Investigators and the Brazilian Research in Intensive Care Network (BRICNet): Effect of flexible family visitation on delirium among patients in the intensive care unit: The ICU visits randomized clinical trial. JAMA 2019; 322:216–228 31310297 10.1001/jama.2019.8766 PMC6635909

[15] Wheeler A Bloch E Blaylock S: Delirium education for family caregivers of patients in the intensive care unit: A pilot study. PEC Innov 2023; 2:100156 37214508 10.1016/j.pecinn.2023.100156 PMC10194211

